# Frequency of Self-Weighing and Weight Change: Cohort Study With 10,000 Smart Scale Users

**DOI:** 10.2196/25529

**Published:** 2021-06-28

**Authors:** Anna-Leena Vuorinen, Elina Helander, Julia Pietilä, Ilkka Korhonen

**Affiliations:** 1 VTT Technical Research Centre of Finland Tampere Finland; 2 Health Sciences Faculty of Social Sciences Tampere University Tampere Finland; 3 Faculty of Medicine and Health Technology Tampere University Tampere Finland

**Keywords:** self-monitoring, self-weighing, weight change, weight loss, normal weight, overweight, obese, temporal weight change

## Abstract

**Background:**

Frequent self-weighing is associated with successful weight loss and weight maintenance during and after weight loss interventions. Less is known about self-weighing behaviors and associated weight change in free-living settings.

**Objective:**

This study aimed to investigate the association between the frequency of self-weighing and changes in body weight in a large international cohort of smart scale users.

**Methods:**

This was an observational cohort study with 10,000 randomly selected smart scale users who had used the scale for at least 1 year. Longitudinal weight measurement data were analyzed. The association between the frequency of self-weighing and weight change over the follow-up was investigated among normal weight, overweight, and obese users using Pearson’s correlation coefficient and linear models. The association between the frequency of self-weighing and temporal weight change was analyzed using linear mixed effects models.

**Results:**

The eligible sample consisted of 9768 participants (6515/9768, 66.7% men; mean age 41.5 years; mean BMI 26.8 kg/m2). Of the participants, 4003 (4003/9768, 41.0%), 3748 (3748/9768, 38.4%), and 2017 (2017/9768, 20.6%) were normal weight, overweight, and obese, respectively. During the mean follow-up time of 1085 days, the mean weight change was –0.59 kg, and the mean percentage of days with a self-weigh was 39.98%, which equals 2.8 self-weighs per week. The percentage of self-weighing days correlated inversely with weight change, *r*=–0.111 (*P*<.001). Among normal weight, overweight, and obese individuals, the correlations were *r*=–0.100 (*P*<.001), *r*=–0.125 (*P*<.001), and *r*=–0.148 (*P*<.001), respectively. Of all participants, 72.5% (7085/9768) had at least one period of ≥30 days without weight measurements. During the break, weight increased, and weight gains were more pronounced among overweight and obese individuals: 0.58 kg in the normal weight group, 0.93 kg in the overweight group, and 1.37 kg in the obese group (*P*<.001).

**Conclusions:**

Frequent self-weighing was associated with favorable weight loss outcomes also in an uncontrolled, free-living setting, regardless of specific weight loss interventions. The beneficial associations of regular self-weighing were more pronounced for overweight or obese individuals.

## Introduction

Self-monitoring of body weight is the cornerstone of behavioral weight loss interventions [1]. Increased awareness gained through self-weighing is expected to trigger a self-evaluation process that involves interpretation of weight data against one’s goal and subsequently results in weight loss–promoting actions [2,3]. Regular self-weighing is consistently associated with weight loss and weight loss maintenance as well as weight gain prevention [2-7]. There is also evidence of a dose-response relationship with more frequent self-weighing and greater weight loss [8-11]. Currently, less is known about self-weighing practices and their effectiveness independent of the context of weight loss interventions.

To date, most studies have investigated self-weighing behaviors through self-reported methods. Self-weighing frequency is often retrospectively reported and evaluated in predefined categories (eg, “daily weighing,” “weekly weighing,” or “monthly weighing”) [9,12,13]. Some studies have determined self-weighing frequency from actual self-weighing data, but further reduced it to a single aggregated frequency value [10,14,15]. Although valuable in research, self-reported data collection methods have their limitations, including selective reporting and susceptibility to recall bias [16,17]. Moreover, single aggregated categories may not reflect long-term weighing practices, as self-weighing frequency tends to change or decline over time [18-20].

Self-monitoring of body weight has become easier and increasingly popular with modern smart scales that automatically transmit weight data to network servers that users can access online or through smart phone applications. As a result, date-stamped and time-stamped weight record data accumulate from a large number of users, providing unique insights into behavioral patterns of weight-tracking individuals. Advantages of using these data in health research include objectivity and accuracy of the information they provide [16,21]. In addition, they provide longitudinal weight measurement data, which are rarely, although increasingly, obtained or used in research. However, while being a strength, self-weighing with smart scales is done by individuals in their daily lives (ie, in uncontrolled, free-living settings), which introduces challenges for the analytics and interpretation of the data. These include the possibility of weight measurement outliers, daily variation, and inadequate background information, among others [21-25].

In our previous work, we demonstrated a dose-response relationship between self-weighing frequency and weight change and an increased risk for weight gain when weight monitoring was stopped [8]. However, the generalization of the findings was limited due to the small sample size (n=40) and the sample consisting of overweight individuals engaged in a workplace health promotion program. The objective of this study was to investigate self-weighing patterns and their association with weight change in a large international sample of smart scale users in their free-living settings. We divided the sample into 3 groups to further study the differences among normal weight, overweight, and obese individuals.

## Methods

### Design and Data Source

The data used in the current study were collected from 10,000 anonymous Withings (Withings Ltd, Paris, France) smart scale users randomly retrieved from the company’s smart scale user database between May 2009 and June 2015. A user was defined as a person who had access to a smart scale and had a user account in which his or her measurements were linked. In the connection of setting up the user account, the user consented to his or her data being used for research purposes by accepting the Terms and Conditions of Withings [26]. For each measurement, the scale automatically saved the weight record and the date stamp and time stamp of the measurement, and the information was further sent to the network servers. Multiple users could share the same scale; however, they were advised to create an individual user account. The scale automatically detected to which user the weight measurement belonged. Users were able to remove weight observations or attach unknown observations to their account manually through a web-based application interface. The data used in the study were pseudonymized before data retrieval and contained all weight measurements attached to a single user account.

In order to study both long-term and short-term weight changes, the smart scale users who fulfilled the following inclusion criteria by June 2015 were eligible for random sampling: engaged with self-weighing for at least 1 year (based on a time difference between the first and the last weight measurements) and had ≥30 weight measurements linked to a user account.

Background information of smart scale users included the following self-reported measures: age in years, height, gender, and location information (ie, country and time zone) that were automatically recorded.

### Study Subjects

In addition to the inclusion criteria used for data extraction, smart scale users who fulfilled the following criteria were included in the current study: age between 18 and 100 years at the time of the first weight measurement, height between 1.40 m and 2.10 m (defined to exclude possible errors in height records that would lead to erroneous BMI values), BMI ≥18.5 kg/m^2^ based on the weight measurements made during the first follow-up week.

### Data Processing

As the weight measurement data were not collected through a structured protocol, but instead accumulated in a free-living setting, the data required preprocessing to exclude possible outlying measurements and multiple intraday measurements from the current analyses. Outliers may arise from situations such as different users using the same devices or due to external, uncontrolled inﬂuences such as exceptionally heavy clothing or carrying an object during self-weighing. We identified and removed the outliers using the following steps. First, all measurements below 30 kg were removed. Second, the outlying measurements were identified with the median absolute deviation method (window=30 samples, threshold=4) and subsequently removed. Third, weight measurements resulting in an unrealistic weight change from the preceding measurement were identified and excluded using a specific algorithm: A weight change was considered unrealistic if it was voted 3 times as an outlier by the 2 preceding and 2 successive measurements, with each measurement encompassing 1 vote. A vote was defined as a difference between the 2 consecutive weight measurements that exceeded the threshold of 3% daily plus 0.1% times day difference between the measurements. The measurements were removed iteratively by removing 1 observation at a time, starting from the outlier that had the highest number of votes and then the highest total sum of absolute weight difference. The process was repeated until no outliers were detected. After removing the outliers, the minimum weight values for each day were identified, and other day duplicates were excluded from the analyses to reduce diurnal variation of weight. Finally, if more than 10 original daily minimum weight values were removed as a result of the outlier detection algorithm (compared to before any processing) or if less than 30 observations were left after preprocessing, the user was excluded from the final analyses.

### Variables

#### Follow-Up Time

The individual follow-up time for each user was defined as the time difference between the first and the last weight measurements.

#### Weight Change

Weight change during the follow-up period was defined as a difference between the mean weight of the last 7 days and the mean weight of the first 7 days (*initial weight*), divided by the initial weight. A similar approach was used by Zheng et al [19]. Negative and positive weight differences indicate weight loss and weight gain, respectively.

#### Temporal Weight Change

Temporal weight change per day was calculated by dividing weight change between 2 consecutive measurements by the corresponding day difference between the measurements.

#### BMI

Height was retrieved from the Withings research database. The baseline BMI was calculated from the self-reported height and the initial weight. Individuals were categorized into 3 categories based on BMI thresholds: normal weight (18.5 kg/m^2^ to <25.0 kg/m^2^), overweight (25.0 kg/m^2^ to <30.0 kg/m^2^), and obese (≥30.0 kg/m^2^).

#### Frequency of Self-Weighing

The self-weighing frequency was defined as a percentage of follow-up days with a weight measurement recorded. We also calculated the number of weight measurements per week defined by dividing the number of all weight measurements with the number of follow-up weeks to be used as a proxy for weekly self-weighing intensity.

For the analysis of temporal weight changes, we used a categorical self-weighing frequency that was defined based on the number of days between the 2 consecutive measurements. The categories were based on commonly used self-weighing categories. The categorization was also motivated by the fact that the majority of measurements were done on a weekly basis or more frequently, and thus, the distribution was strongly skewed. The self-weighing categories were as follows: daily — 0 days between 2 consecutive weight measurements; every other day — 1 day between 2 consecutive weight measurements; 2-4 times per week — 2 days between 2 consecutive weight measurements; 1-2 times per week — 3-7 days between 2 consecutive weight measurements; every other week — 8-14 days between 2 consecutive weight measurements; monthly — 14-29 days between 2 consecutive weight measurements; less than monthly — ≥30 days between consecutive measurements.

#### Break in Self-Weighing

A break in self-weighing was defined as ≥30 days between 2 consecutive weight measurements. The weight change during the break was calculated as a difference between the last preceding weight measurement and the first weight measurement after the break. If the user had multiple breaks, the mean weight change and the mean duration of all breaks were calculated.

#### Time of the Day and the Weekday

To control time effect, weight measurements were each categorized based on their time stamp to either a morning (measurement made between 5 am and 1 pm) or an evening (measurement made between 1 pm and the 5 am the next day) measurement. We further defined a combination variable accounting for time stamps of the present and the previous weight measurement with 4 possible categories: morning-morning, morning-evening, evening-evening, evening-morning. This variable was used in the analyses of temporal weight changes. Similarly, as weekday is shown to affect weight variation [24,27], we defined the weekday of the weight measurement and used the variable in the analyses of temporal weight change.

### Statistical Analyses

Participant characteristics are presented using means (SDs) or frequencies and percentages. Characteristics were compared across the 3 BMI groups using a Chi-square test or analysis of variance or Kruskall Wallis test if the distributions were strongly skewed. Similar comparisons across the BMI groups were made among participants who had a break in self-weighing.

The Pearson correlation coefficient (r) between weight change (kg) and the frequency of self-weighing, measured as a percentage of follow-up days with a weight measurement, was calculated for the whole study population and separately for each BMI group. We then built linear models with an interaction term between the BMI group and the frequency of self-weighing to investigate whether the association was different across the BMI groups. The adjusted models were further performed including the following covariates: sex, age, initial BMI, and the duration of the follow-up.

In the secondary analyses, we focused on temporal weight changes and analyzed the association between temporal weight change and the corresponding day difference by using linear mixed effect (LME) models. Day difference between 2 consecutive weight measurements was added in the model as a fixed effect and participants as a random effect. Each participant was allowed to have a subject-specific intercept and a slope. The adjusted analyses included the following covariates: sex, age, initial BMI, weekday, and time of the day combination. Temporal weight change was also modeled using the categorized self-weighing frequency variable as a dependent variable and further adding the interaction between the self-weighing category and BMI group in the model as a fixed effect.

Statistical analyses were carried out with R version 3.6.2 [28] using dplyr [29] and nlme [30] packages. An alpha level of 0.05 was used in all statistical analyses, and all statistical tests were two-sided.

## Results

### Participant Inclusion and Background Characteristics

**Figure 1:**
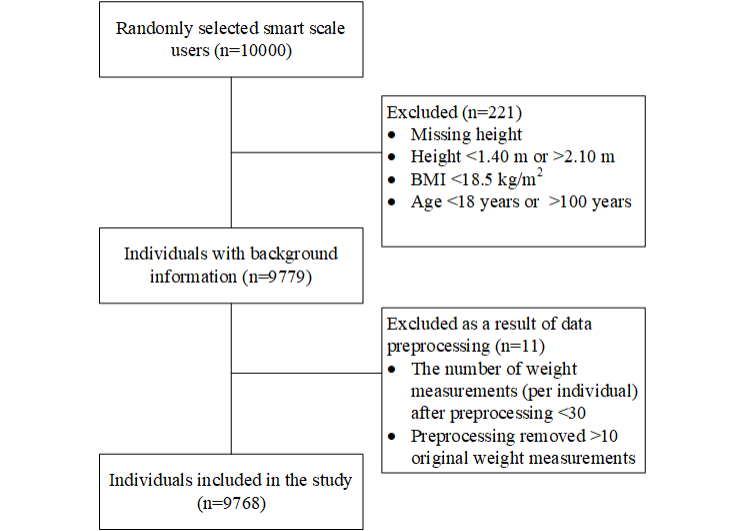
Participant inclusion and data prepreprocessing.

The participant inclusion and data preprocessing flow is depicted in Figure 1. Of the 10,000 smart scale users, 9768 (97.7%) were included in the study. In total, participants recorded 4,230,928 weight measurements.

Participants’ background characteristics and self-weighing–related information are summarized in Table 1. The majority (6515/9768, 66.7%) were male. The mean age was 41.52 years, and the mean BMI was 26.78 kg/m^2^. Of all participants, 4003 (4003/9768, 42.0%) were of normal weight, 3748 (3748/9768, 38.4%) were overweight, and 2017 (2017/9768, 20.7%) were obese at the beginning of the follow-up. The proportion of male participants was higher in the overweight and obese groups than among the normal weight group (2969/3748, 79.2%; 1551/2017, 76.9%; 1995/4003, 49.8%, respectively; *P*<.001). Of the included participants, 4837 (4837/9768, 49.5%) were from Europe, 3744 (3744/9768, 38.3%) from the Americas, 887 (887/9758, 9.1%) from Asia, and the rest (300/9768, 3.0%) from other continents. The most represented countries were the United States (3332/9768, 34.1%), Germany (1230/9768, 12.6%), France (811/9768, 8.3%), Japan (600/9768, 6.1%), and the United Kingdom (585/9768, 6.0%).

The mean duration of the follow-up was 1085 days, which did not differ across the 3 BMI groups (*P=*.35). The median number of self-weighs was 352, which equals 2.80 weight measurements per week. The highest self-weighing intensity was seen among overweight individuals; however, the differences between the groups were small, although statistically significant, varying from 38.99% among obese individuals to 40.86% among overweight individuals (*P=*.006). The mean percentage days with a weight measurement was 39.98%.

**Table 1:** Background characteristics and self-weighing–related information for smart scale users.

Characteristic	All participants (n=9768)	Normal weight group (n=4003)	Overweight group (n=3748)	Obese group (n=2017)	Statistic for the difference between the BMI groups	*P* value
Gender (male), n (%)	6515 (66.7)	1995 (49.8)	2969 (79.2)	1551 (76.9)	*χ^2^_2_*=871.15	<.001
Age (years), mean (SD)	41.52 (11.08)	40.07 (10.86)	42.33 (11.07)	42.88 (11.21)	*F_2,9765_*=60.24	<.001
Weight (kg), mean (SD)	82.01 (19.03)	67.01 (9.96)	84.74 (9.86)	106.71 (17.48)	N/A^a^	N/A
BMI (kg/m^2^), mean (SD)	26.78 (5.01)	22.55 (1.68)	27.20 (1.42)	34.38 (4.32)	N/A	N/A
Follow-up time (days), mean (SD)	1085 (420.17)	1086 (422.65)	1090 (419.16)	1073 (417.08)	*F_2,9765_*=1.05	.35
Total number of weight measurements, median (IQR)	352.00 (197.00 to 591.00)	345.00 (190.00 to 588.50)	371.00 (213.75 to 604.00)	338.00 (189.00 to 571.00)	*KW_2_^b^*=18.59	<.001
Percentage of days with a weight measurement, mean (SD)	39.98 (22.80)	39.65 (23.41)	40.86 (22.24)	38.99 (22.54)	*F_2,9765_*=5.09	.006
Weight measurements per week, mean (SD)	2.80 (1.60)	2.78 (1.64)	2.86 (1.56)	2.73 (1.58)	*F_2,9765_*=5.09	.006
Weight change (kg), mean (SD)	–0.59 (6.99)	0.78 (4.38)	–0.58 (5.96)	–3.35 (11.01)	*F_2,9765_*=246.00	<.001
Weight change (%), mean (SD)	–0.37 (7.64)	1.20 (6.57)	–0.68 (7.09)	–2.93 (9.61)	*F_2,9765_*=209.4	<.001
Maximum weight change (kg), median (IQR)	–4.97 (–8.52 to –2.76)	–3.49 (–5.42 to –2.03)	–5.81 (–8.97 to –3.38)	–9.33 (–15.73 to –4.99)	*KW_2_*=1827.00	<.001

^a^N/A: not available because no statistical test was performed to test differences between the groups.

^b^KW: Kruskal-Wallis.

### Frequency of Self-Weighing and Weight Change

During the mean follow-up of 1085 days, the participants lost 0.59 kg of their initial weight (Table 1). The mean percentage weight change was –0.37%. Weight changes varied between the BMI groups, with an increase of 0.78 kg in the normal weight group and decreases of –0.58 kg and –3.35 kg in the overweight and obese groups, respectively.

There was a low, statistically significant, correlation between the frequency of self-weighing, measured as the percentage of self-weighing days, and weight change (*r*=–0.111, *P*<.001). The correlation was stronger in the highest BMI groups: *r*=–0.100 (*P*<.001) for normal weight, *r*=–0.125 (*P*<.001) for overweight, and *r*=–0.148 (*P*<.001) for obese. The linear model, including the interaction term between the BMI group and the frequency of self-weighing, showed a statistically significant interaction for the overweight and obese groups and the frequency of self-weighing (*β*=–0.015, *P=*.03 and *β*=–0.053, *P*<.001, respectively); see Table S1 in Multimedia Appendix 1 for all model coefficients. The result indicates that, when compared to the normal weight group, the association between the frequency of self-weighing and weight change was different among obese and overweight individuals. Similar parameter estimates were obtained from the adjusted models (*β*=–0.017, *P=*.01 for the overweight group and *β*=–0.057, *P*<.001 for the obese group).

**Figure 2:**
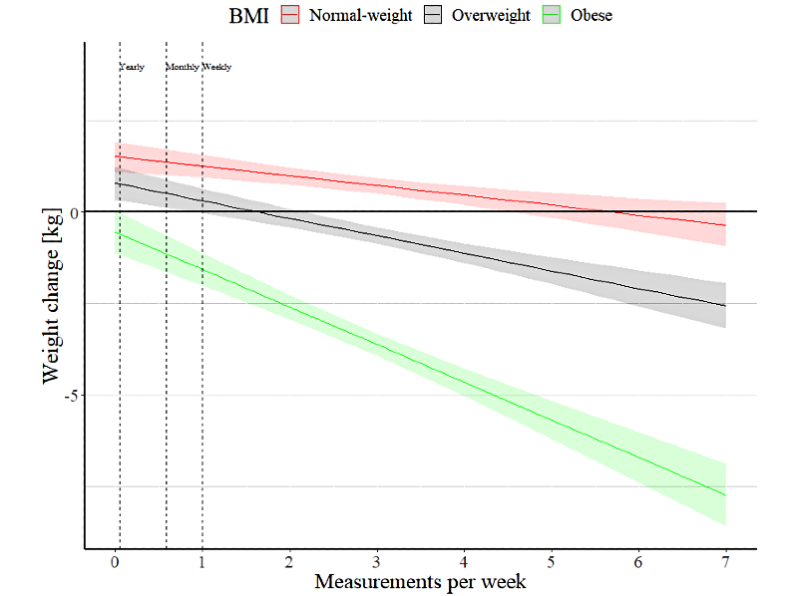
Weight change during the study follow-up predicted by the number of weight measurements per week.

Figure 2 shows the weight change (kg) in the 3 BMI groups predicted by the weekly weighing frequency. A slightly steeper slope was seen in the obese group as compared to the normal weight group illustrating the interaction. A higher frequency of self-weighing was associated with bigger weight loss in the obese group. The magnitude of the interaction was smaller in the overweight group. The figure shows that weighing oneself at least once a week was associated with negative weight change in the obese group, whereas in the overweight and normal weight groups, the confidence intervals overlap the 0 line or remain above it. It seems that a self-weighing frequency of 2-3 times per week, or more frequently, was associated with negative weight change in the overweight group. However, in the normal weight group, the upper limit of the 95% confidence interval was not below 0 at all.

### Temporal Weight Change and Self-Weighing Frequency

After calculating the temporal weight change variable, we excluded measurements that exceeded the threshold of 20% weight change per day (n=35). The number of individuals in the analyses remained at 9768. The LME model showed that the number of days between 2 consecutive weight measurements was a significant predictor of temporal weight change (*β*=0.001, *P*<.001); weight increased by the number of days between the weight measurements. The adjusted model did not alter the parameter estimate (*β*=0.001, *P*<.001).

**Figure 3:**
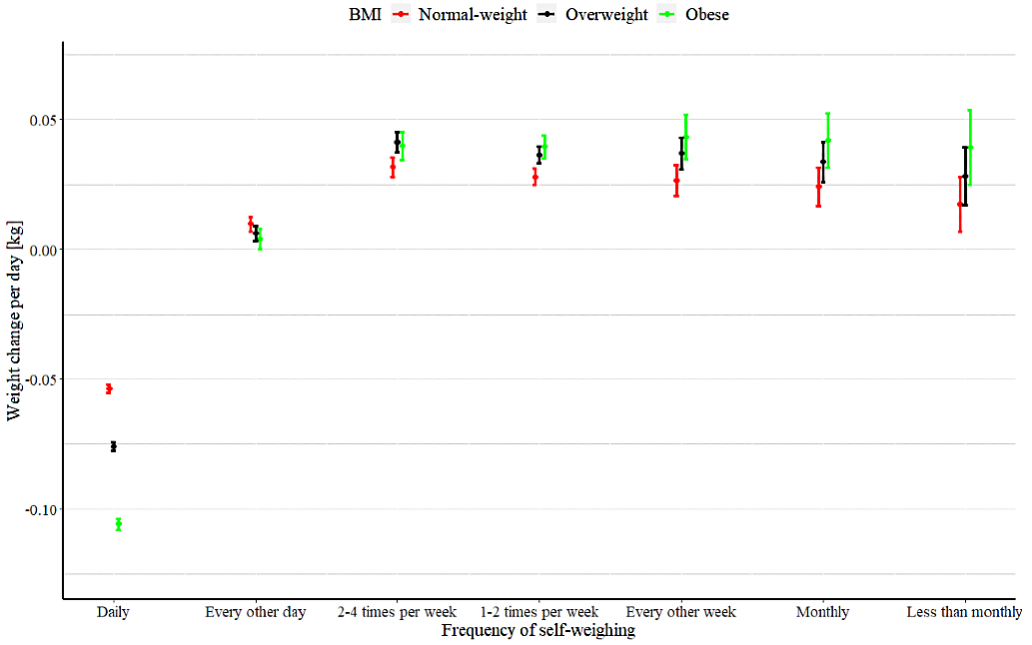
Weight change per day predicted by the interaction between the frequency of self-weighing and BMI group.

The mean weight changes per day in the self-weighing frequency categories were as follows: daily, –0.058 kg; every other day, 0.012 kg; 2-4 times per week, 0.036 kg; 1-2 times per week, 0.028 kg; every other week, 0.026 kg; monthly, 0.022 kg; less than monthly, 0.016 kg. Temporal weight changes in each category were significantly different from the “daily” value, which was used as a reference category (Multimedia Appendix 1, Table S2). Figure 3 shows the interactions between self-weighing categories and the BMI groups. The parameter estimates of the model are shown in Multimedia Appendix 1 Table S3. Only daily self-weighing was associated with a negative temporal weight change per day in all BMI groups.

### Breaks in Self-Weighing

A majority of the participants (7085/9768, 72.5%) had at least one break in self-weighing of ≥30 days during the follow-up (Table 2). In the normal weight, overweight, and obese groups, 73.5% (2941/4003), 70.7% (2648/3748), and 74.2% (1496/2017), respectively, had at least one break. The median number of breaks was 3 with an average duration of 58.83 days. During the break, the participants gained 0.85 kg on average. The median increase was bigger in the obese group (1.37 kg) than in the overweight (0.93 kg) and normal weight (0.58 kg) groups (*P*<.001). The Spearman correlation coefficients between the duration of the break and weight change (kg) were *ρ*=0.153 (*P*<.001) among all participants and *ρ*=0.090 (*P*<.001), *ρ*=0.161 (*P*<.001), and *ρ*=0.245 (*P*<.001) in the normal weight, overweight, and obese groups, respectively.

**Table 2:** The numbers of breaks in self-weighing, their durations, and the subsequent weight change among all study participants who had a break from self-weighing and in the 3 BMI groups.

Characteristic	All who had a break from self-weighing (n=7085)	Normal weight (n=2941)	Overweight (n=2648)	Obese (n=1496)	Kruskal Wallis statistic for the difference between the BMI groups	*P* value
Number of breaks, median (IQR)	3 (2 to 5)	3 (2 to 5)	3 (1 to 5)	3 (2 to 6)	8.48 (df=2)	.01
Duration of the break (days), median (IQR)	58.83 (44.00 to 86.30)	58.33 (44.00 to 85.00)	57.75 (43.62 to 85.55)	61.20 (45.36 to 90.00)	11.44 (df=2)	.003
Weight change (kg), median (IQR)	0.85 (0.03 to 1.92)	0.58 (–0.13 to 1.46)	0.93 (0.06 to 1.99)	1.37 (0.38 to 2.61)	247.32 (df=2)	<.001
Weight change (%), median (IQR)	1.05 (0.04 to 2.30)	0.87 (–0.18 to 2.20)	1.10 (0.08 to 2.32)	1.30 (0.36 to 2.46)	58.54 (df=2)	<.001

## Discussion

### Principal Findings

This study extends the well-established relationship between frequent self-weighing and favorable weight loss outcomes beyond specific weight loss interventions by using a large international sample of 9768 long-term smart scale users, tracking their weight in uncontrolled, free-living settings. We found that the intensity of self-weighing was inversely associated with weight change, and the beneficial associations of frequent self-weighing were more pronounced in obese and overweight individuals as compared to normal weight individuals. Daily self-weighing in particular was associated with weight loss.

During the mean follow-up time of 1085 days, the participants measured their weight 2.8 times per week, which is notably higher than what is reported in the general population of the United States [15]. However, a comparable self-weighing intensity was reported in the Heart eHealth (HeH) study [19], a prospective observational cohort study on health behaviors and cardiovascular risk. Similarly, the HeH study reflects free-living behaviors, as study participants did not receive any recommendations for self-weighing practices, but those who owned smart scales connected them to the HeH study account.

While the self-weighing frequency was rather high, more than two-thirds of the study participants also had at least one longer break in self-weighing (≥30 days) during the follow-up. The break in self-weighing was associated with increased weight, with a dose-response relationship with the length of the break: The longer the break, the more weight tended to increase. Moreover, weight gain following the break in self-weighing was largest among obese individuals (+1.37 kg) and overweight individuals (+0.93 kg) when compared to normal weight individuals (+0.58 kg), while the average length of the break was rather similar between all 3 BMI groups (varying between 58 days and 61 days). Similar findings were also reported by VanWormer et al [12], who found that monthly self-weighers actually gained weight during the 24-month follow-up. These results suggest that regular self-weighing might be of particular importance in terms of promoting weight loss for individuals with excess weight, possibly by helping them attain weight management–promoting behaviors.

The correlation between frequent self-weighing and weight loss supports the beneficial effect of weight monitoring and, as such, is in line with other studies [3,8,9,11,13]. The association seemed to be stronger in individuals who needed it most, namely obese and overweight individuals, as seen earlier [12]. The motivation for weight tracking in individuals who do not have excess weight is likely different, as they may not pursue weight loss, which might at least partly explain the lower correlation in the normal weight group. It should be noted that the correlation coefficients were generally very low, which indicates that self-weighing explains relatively little of the weight change variation. Other factors, such as long-term goals, motivation for self-weighing, and behavioral factors as well as diet and physical activity, may have stronger effects on weight loss efforts, although weight-loss–promoting practices are important. It has also been suggested that the consistency of self-weighing is more important than the frequency of self-weighing [31].

As self-weighing practices tend to vary and decline over time [13,20,32], we analyzed temporal weight changes using consecutive weight measurements and the corresponding day differences to investigate the short-term associations. These results were consistent with aggregated data showing a significant relationship between weight change and the corresponding day difference. The higher the number of days between self-weighs, the more weight increased. In these analyses, we used weight change per day as an independent variable, which adjusted the weight change by the number of days between the measurements to control for the possibility of bigger weight changes during longer breaks. When using the self-weighing categories, we found that only daily self-weighing was associated with weight loss in all BMI groups, whereas weighing oneself every other day or less frequently was associated with unchanged or increased weight. Interestingly, weighing every other day was associated with increased weight in normal weight and overweight groups, whereas in obese individuals, it was associated with unchanged weight. Consistent with our findings, it has been shown that daily weighing is associated with greater weight loss [33], even when compared to weighing oneself 5 days of the week [11]. However, not all studies have reported the superiority of daily self-weighing over weekly monitoring [5]. The positive impact of daily weighing vs weighing oneself every other day or on a weekly basis in our observational study should be interpreted with caution. It is possible that the differences between daily and every-other-day categories reflect an ongoing weight loss process rather than daily self-weighing being remarkably more effective than weighing oneself every other day.

### Limitations

The findings of this study should be interpreted in the light of the following limitations. First, as an observational study, a causal relationship between self-weighing and weight change cannot be established. Efforts at self-weighing could promote weight loss or be a consequence of favorable weight change. In fact, a recent study by Frie et al [25] found that declining motivation for weight control and possible difficulties with making use of negative weight feedback, the Ostrich effect, precede a break in self-weighing. Thus, it might not be the break in self-weighing that increases the risk of weight gain, but that the decline in self-weighing behavior is a result of weight gain. In line with this, Tanenbaum et al [34] showed that calorie intake on a given day increased the risk of not self-weighing the following day; in another study, a reduction in BMI was independently associated with greater engagement with self-weighing [21]. In light of those studies, the findings of the current study may also indicate that the relationship describes the process of weight loss rather than promotes it.

Second, participants’ background information was limited to demographic variables, preventing us from controlling for important variables, such as goals for weight management or the underlying motivation for using the smart scale. For example, among normal weight individuals, it might be that they did not pursue weight loss but rather weight (loss) maintenance, which might explain a weaker correlation seen in this subgroup. On the other hand, obese individuals might be more likely to use the scale for weight loss purposes.

The third limitation is the selection bias. Our sample consisted of individuals who were predominantly men (66.7%), had excess weight (BMI of 26.8 kg/m^2^), and, importantly, were engaged with regular self-monitoring of weight for a longer time period. The sample is not representative of the general population; Sperrin et al [21], who used comparable data from the same company, showed that the traits of those self-weighing individuals differed from the general British population. Participants’ characteristics in our study were quite similar to the study by Sperring et al [21], although they required only one weight measurement per subject and used data from a single country. Another study found that individuals using smart scales in wireless settings were mostly well educated, white, and male [19]. Moreover, due to the inclusion criteria we applied for sampling, our cohort may not be representative of smart scale users, as those who used the scale for a shorter period (less than a year) and made fewer than 30 measurements were excluded. Nevertheless, this was a large international cohort including almost 10,000 individuals from 109 different countries who provided self-weighed measurements for approximately 3 years. These individuals were not participating in a specific weight loss program, but the data reflect self-weighing behaviors in real-world settings. We consider the overrepresentation of men as a strength of this study. Typically, participants in weight management interventions are women. Thus, the findings of this study shed light on self-weighing patterns and weight loss attempts by men, who are less investigated. Moreover, together with other smart scale–based studies, the findings indicate that including technology to support weight loss may have the potential to appeal to men to participate in weight control trials.

Fourth, measuring longitudinal changes in self-weighing behaviors remain a challenge. Aggregating data over multiple days and weeks, as we did in the first analysis, does not account for changing behavioral patterns. In an attempt to tackle the challenge, we investigated short-term weight changes by analyzing consecutive weight measurements. However, these results may have been affected by the underlying trend of the weight loss process; individuals who lose weight may be more likely to weigh themselves daily, and thus, even 1-day breaks from the daily self-weighing may appear as short-term weight gains. As there were some differences in results obtained with these 2 analysis approaches, future studies should note that the length of the follow-up might affect the results when investigating how the frequency of self-weighing affects weight loss. Another alternative for the analysis of similar data to ours would be to identify slots with unchanged self-weighing pattern as was done by Frie et al [25] and to analyze associated weight changes.

### Conclusions

This study showed that frequent self-weighing is associated with favorable weight loss outcomes also in an uncontrolled, free-living setting, regardless of specific weight loss interventions. The positive association of regular self-weighing was more pronounced for individuals who needed it most, namely overweight and obese individuals. We found that daily self-weighing in particular was associated with weight loss, whereas breaks of 30 days or longer were associated with increased weight. However, although, there was a dose-response relationship between self-weighing intensity and weight change, the correlation remained low, indicating that self-weighing explains only a small fraction of the variations in weight. Yet, our results underscore the evidence that missing self-weighing data do not occur randomly and might be a sign of risk for weight gain.

## Appendix

Multimedia Appendix 1 Supplementary data.

## Abbreviations

HeH: Heart eHealth study
LME: linear mixed effect
