# Supplementary material for: Frequency of Self-Weighing and Weight Change: Cohort Study With 10,000 Smart Scale Users
Source: J Med Internet Res. 2021 Jun 28;23(6):e25529. doi: 10.2196/25529 (PMC8277333; doi:10.2196/25529)
Supplement: Multimedia Appendix 1 [file jmir_v23i6e25529_app1.docx]

## Multimedia Appendix 1

**Table S1**. β-coefficients and their P-values for the linear model where weight change during the study follow-up was predicted by the interaction between the percentage of follow-up days with a self-weigh and the BMI group.

|  | **β** | **P-value** |
| --- | --- | --- |
| Intercept | 1.519 | < .001 |
| Percentage of self-weighing days | -0.019 |  |
| Normal-weight | reference category | < .001 |
| Overweight | -0.724 | .02 |
| Obese | -2.060 | < .001 |
| Percentage of self-weighing days: overweight | -0.015 | .03 |
| Percentage of self-weighing days: obese | -0.053 | < .001 |

**Table S2.** β-coefficients and their P-values for the linear mixed effects-model where temporal weight change is predicted by the categorical self-weighing variable.

|  | **β** | **P-value** |
| --- | --- | --- |
| Intercept | -0.074 | < .001 |
| Daily | reference category |  |
| Every other day | 0.080 | < .001 |
| 2-4 times per week | 0.111 | < .001 |
| 1-2 Times per week | 0.107 | < .001 |
| Every other week | 0.108 | < .001 |
| Monthly | 0.105 | < .001 |
| Less than monthly | 0.100 | < .001 |

**Table S3.** β coefficients and their P-values for the linear mixed effects-model where temporal weight change is predicted by the interaction between the categorical self-weighing variable and the BMI group.

|  | **β** | **P-value** |
| --- | --- | --- |
| Intercept | -0.054 | < .001 |
| Normal-weight | reference category |  |
| Overweight | -0.022 | < .001 |
| Obese | -0.052 | < .001 |
| Daily | reference category |  |
| Every other day | 0.063 | < .001 |
| 2-4 times per week | 0.085 | < .001 |
| 1-2 times per week | 0.081 | < .001 |
| Every other week | 0.080 | < .001 |
| Monthly | 0.078 | < .001 |
| Less than monthly | 0.071 | < .001 |
| Every other day: overweight | 0.019 | < .001 |
| 2-4 times per week: overweight | 0.032 | < .001 |
| 1-2 times per week: overweight | 0.031 | < .001 |
| Every other week: overweight | 0.033 | < .001 |
| Monthly: overweight | 0.032 | < .001 |
| Less than monthly: overweight | 0.033 | < .001 |
| Every other day: obese | 0.047 | < .001 |
| 2-4 times per week: obese | 0.060 | < .001 |
| 1-2 times per week: obese | 0.064 | < .001 |
| Every other week: obese | 0.069 | < .001 |
| Monthly: obese | 0.070 | < .001 |
| Less than monthly: obese | 0.074 | < .001 |
